# Case Report: Synovial sarcoma with diffuse myxoid stroma and complete absence of epithelial differentiation in the extremity

**DOI:** 10.3389/fonc.2026.1846272

**Published:** 2026-05-29

**Authors:** Tomohiro Miyazaki, Naoki Oike, Takashi Ariizumi, Yudai Murayama, Akira Ogose, Hideaki Sugino, Mai Nakamura, Shuhei Kondo, Yusuke Tani, Hajime Umezu, Hiroyuki Kawashima

**Affiliations:** 1 Department of Orthopedics, Niigata University Graduate School of Medical and Dental Sciences, Niigata, Japan; 2 Department of Orthopedic Surgery, Uonuma Institute of Community Medicine, Niigata University Medical and Dental Hospital, Minami-Uonuma, Japan; 3 Department of Pathology, Niigata University Graduate School of Medical and Dental Sciences, Niigata, Japan

**Keywords:** comprehensive genomic profiling, extraskeletal myxoid chondrosarcoma, molecular analysis, myxoid sarcoma, synovial sarcoma

## Abstract

Synovial sarcoma is a translocation-associated soft-tissue sarcoma defined by the SS18–SSX fusion gene and typically exhibits biphasic or monophasic histology with at least focal epithelial marker expression. However, rare cases with diffuse myxoid stroma may closely mimic other myxoid sarcomas, which can pose a diagnostic challenge. A 61-year-old woman presented with a gradually enlarging mass on the posterior aspect of the right thigh. Magnetic resonance imaging revealed a 55-mm soft tissue tumor with heterogeneous high signal intensity on T2-weighted images and partial contrast enhancement. Core needle biopsy demonstrated atypical spindle to round cells within a prominent myxoid background. Immunohistochemically, cytokeratin was negative and SOX9 was diffusely positive, raising suspicion for extraskeletal myxoid chondrosarcoma. The patient underwent marginal excision followed by postoperative radiotherapy; however, lung metastasis and local recurrence subsequently developed. Comprehensive genomic profiling identified an SS18–SSX1 fusion gene, and the diagnosis of synovial sarcoma was confirmed by fluorescence *in situ* hybridization and reverse transcription polymerase chain reaction in both the primary and recurrent tumors. RNA sequencing further verified the SS18–SSX1 fusion and demonstrated no additional pathogenic or clinically relevant fusion transcripts. Histologically, the tumor consistently exhibited a diffuse myxoid stroma with a focal reticular pattern, closely resembling extraskeletal myxoid chondrosarcoma. It lacked epithelial marker expression, including cytokeratin and epithelial membrane antigen (EMA), throughout the disease course. To our knowledge, this case represents a rare presentation of synovial sarcoma arising in the extremity with diffuse myxoid stroma and complete absence of epithelial marker expression, with the diagnosis confirmed by molecular identification of the specific fusion transcript. This case highlights that synovial sarcoma can exhibit a myxoid phenotype and may lack epithelial marker expression, both of which can complicate the diagnosis. It also underscores the importance of integrating molecular analyses, particularly fusion-oriented genomic testing, for accurate diagnosis in challenging soft-tissue tumors.

## 1 Introduction

Synovial sarcoma (SS) is an aggressive soft-tissue malignancy that accounts for approximately 5–10% of adult sarcomas (1). It most commonly occurs in the periarticular soft tissues of the extremities of adolescents and young adults (2). SS is defined by a pathognomonic chromosomal translocation t(X;18)(p11;q11), resulting in an SS18::SSX fusion oncogene (3). Histologically, SS typically exhibits a monophasic or biphasic morphology with epithelial differentiation (3). However, variants with abundant myxoid or mucinous extracellular matrix are rare. In such cases, a diffuse myxoid background can obscure the classic SS morphology, mimicking other myxoid sarcomas or carcinomas (4, 5). These reports highlight that recognizing myxoid/mucinous SS is crucial to avoid misdiagnosis and that immunohistochemistry and molecular tests are essential (4, 5). Herein, we report a diagnostically challenging case of SS with a diffuse myxoid stroma and negative epithelial marker expression.

## 2 Case presentation

### 2.1 Clinical summary

**Figure 1:**
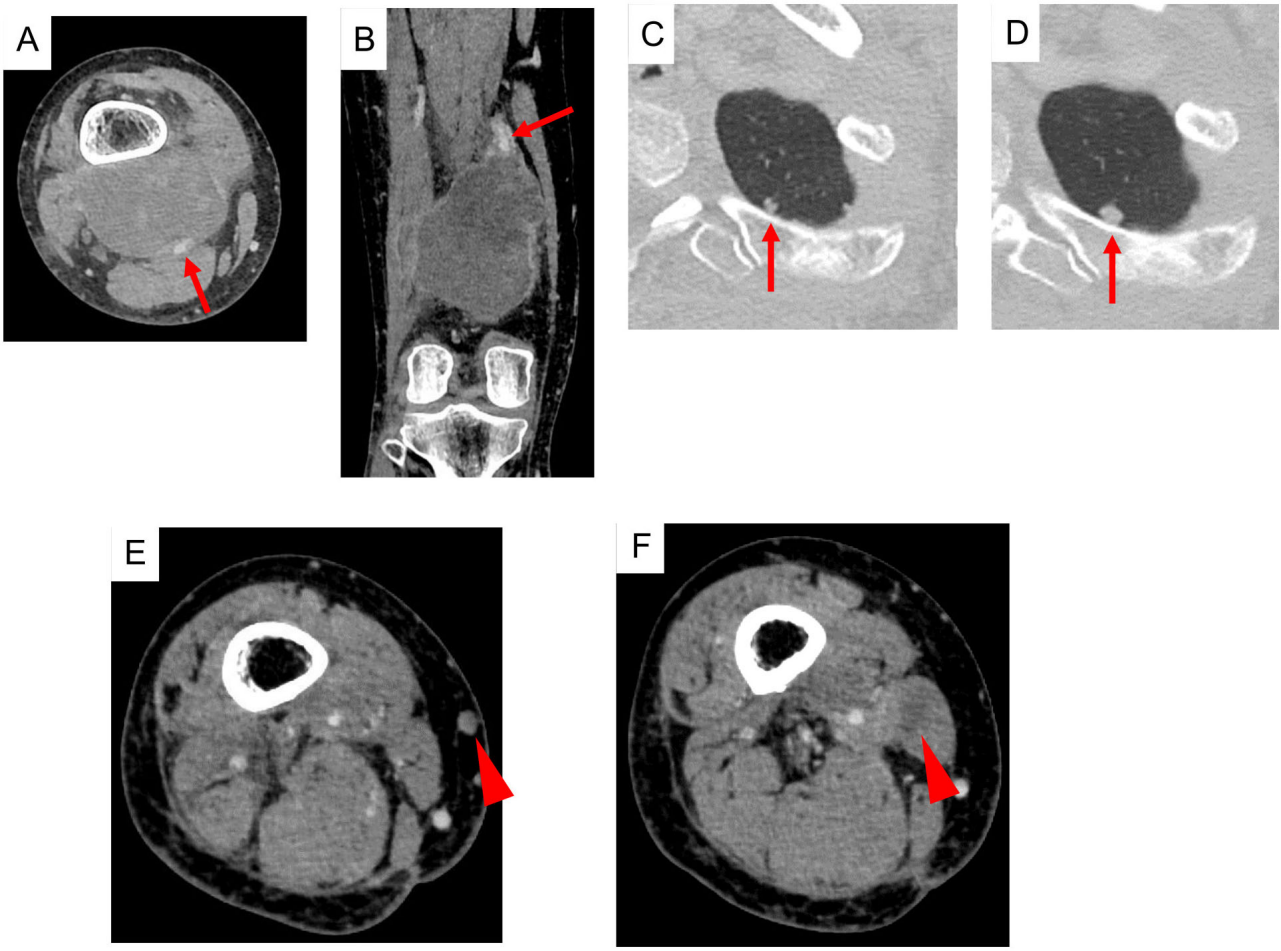
Radiological findings of the tumor. A tumor lesion was observed adjacent to the posterior aspect of the femur. The mass was in contact with the femoral artery, indicated by the arrows. **(A, B)** A left pulmonary lesion was detected 15 months after surgery **(C)** and gradually increased in size at 40 months after surgery **(D)**, as indicated by the arrows. Moreover, at 40 months after surgery, tumor lesions (recurrent tumors) appeared in the subcutaneous tissue **(E)** and intramuscular layer of the right thigh **(F)**, as indicated by the arrowheads.

A 61-year-old woman presented with a gradually enlarging mass on the posterior aspect of the right thigh. She had no significant medical or trauma history. Physical examination revealed a palpable mass on the posterior aspect of the right thigh. Laboratory findings were unremarkable. Computed tomography (CT) was performed for both local and systemic assessments, and magnetic resonance imaging (MRI) was performed to further evaluate the characteristics of the mass. CT revealed a soft-tissue tumor measuring 55 mm in the greatest dimension adjacent to the femur (**Figures 1A, B**), demonstrating partial internal contrast enhancement. No evidence of distant metastasis was identified at the time of the initial evaluation. On MRI, the lesion was isointense to mildly hyperintense relative to the muscle on T1-weighted images, and exhibited heterogeneous high signal intensity on T2-weighted images, with partial enhancement following gadolinium administration (**Supplementary Figure 1**). Core needle biopsy revealed spindle-to-round atypical cells on a myxoid background. Immunohistochemically, the tumor was diffusely positive for SOX9, raising the possibility of extraskeletal myxoid chondrosarcoma (EMC), although a definitive histological subtype could not be established. Given the suspicion of EMC, neoadjuvant chemotherapy was not administered, and surgical management was selected. Wide resection was considered; however, this approach would have required combined resection of the femoral artery adjacent to the tumor, as well as management of the femur. Considering the invasiveness of the procedure and the patient’s activities of daily living, a marginal excision was performed, followed by postoperative radiotherapy (60 Gy in total). The tumor was firmly adherent to the femoral vessels and was particularly inseparable from the femoral vein; therefore, en bloc resection of the femoral vein was performed. At that time, EMC was considered the leading differential diagnosis, and because EMC is generally regarded as insensitive to chemotherapy (6), adjuvant chemotherapy was not pursued following resection of the primary tumor. However, a small indeterminate nodule was detected in the left lung 15 months after surgery (**Figure 1C**) and was subsequently monitored with radiological follow-up. The lesion showed gradual growth over time and was strongly suspected to represent pulmonary metastasis by 25 months. At that time, pulmonary biopsy and systemic therapy were discussed with the patient; however, the patient opted for continued observation, and radiological follow-up was continued. CT performed 40 months after surgery revealed further enlargement of the pulmonary metastasis (**Figure 1D**). Simultaneously, local recurrence was suspected in the subcutaneous tissue and intramuscular layer of the right thigh (**Figures 1E, F**). The pulmonary lesion was not histologically confirmed but was clinically considered metastatic based on radiological progression. To confirm the diagnosis and determine the subsequent treatment strategy, the subcutaneous tumor in the right thigh was resected, and the specimen was subjected to pathological evaluation and comprehensive genomic profiling (CGP). Although no druggable genomic alterations were identified, detection of the SS18–SSX1 fusion gene led to the diagnosis of SS. The patient subsequently received three cycles of doxorubicin; however, both the pulmonary lesion and the local disease showed progression. Pazopanib was then initiated, and over the subsequent three months, tumor progression remained relatively slow; therefore, the treatment is ongoingamid. The clinical course of this patient is summarized in **Supplementary Table 1**.

### 2.2 Pathological findings

**Figure 2:**
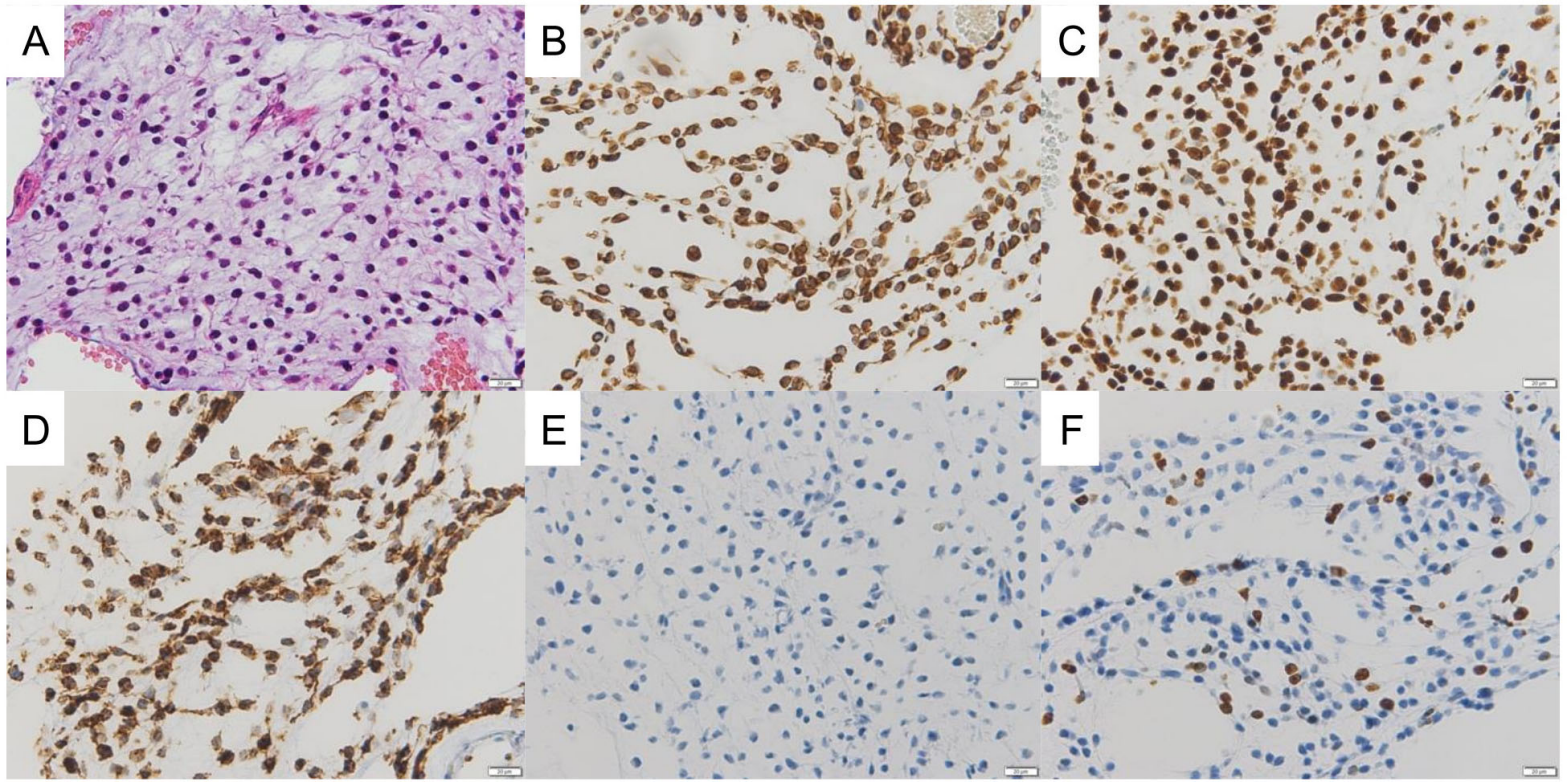
Histological findings of the biopsy specimen. The biopsy specimen showed proliferation of short spindle to round tumor cells within a faintly basophilic myxoid stroma, accompanied by nuclear atypia with minimal pleomorphism **(A)**. Immunohistochemically, the tumor cells were diffusely positive for vimentin **(B)**, SOX9 **(C)**, and CD56 **(D)**, and negative for cytokeratin (keratin mix) **(E)**. The Ki-67 labeling index was approximately 20% **(F)**. Original magnification ×200 for **(A–F)**.

**Figure 3:**
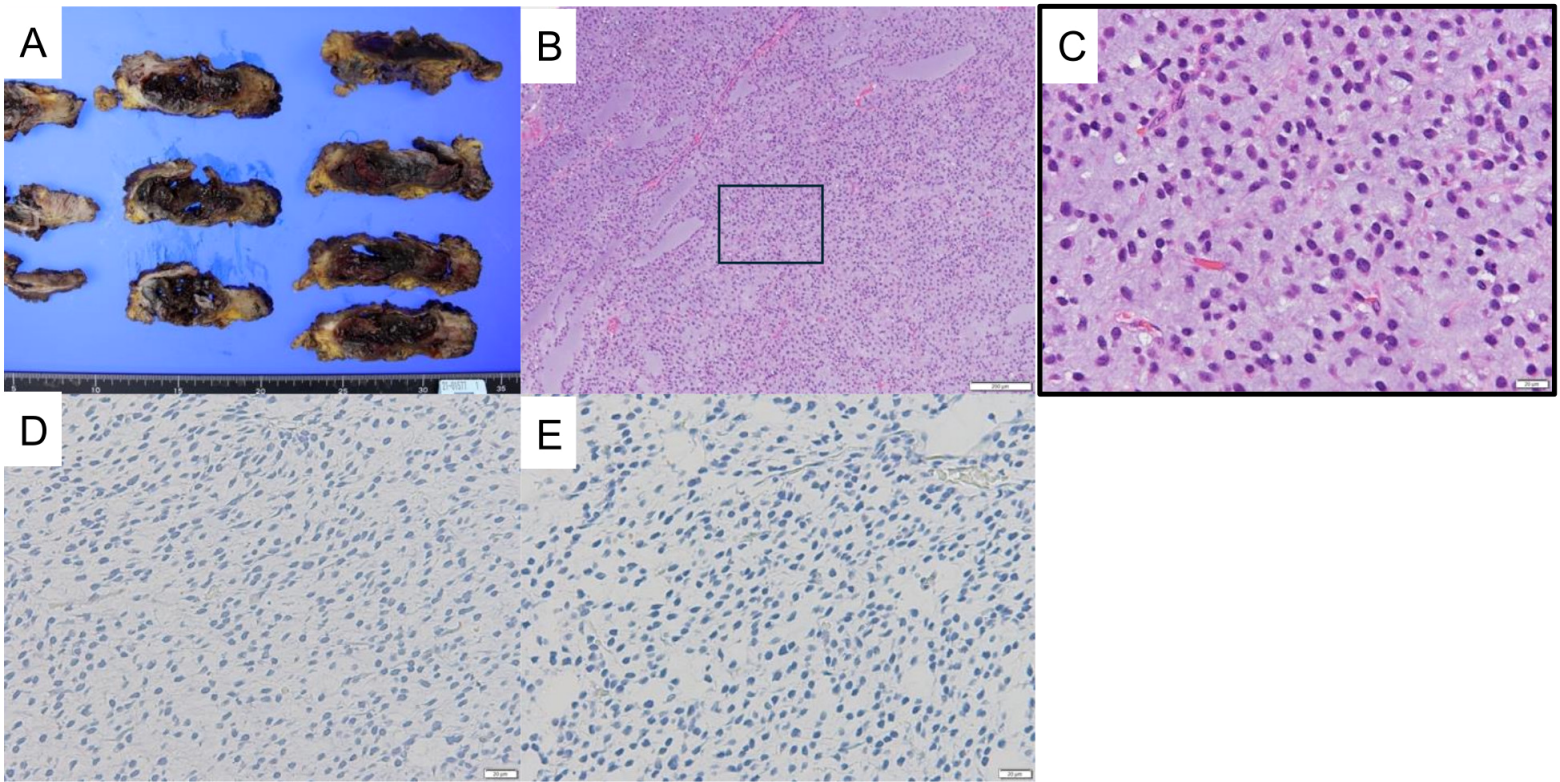
Gross and histological findings of the resected tumor. The resected tumor demonstrated intratumoral hemorrhage and necrosis **(A)**. Histologically, the resected specimen showed proliferation of short spindle-to-round tumor cells in the myxoid stroma **(B, C)**. Immunohistochemically, the tumor cells were negative for EMA **(D)** and AE1/AE3 **(E)**. A, gross photograph; Original magnification ×40 for B and ×200 for **(C–E)**.

**Figure 4:**
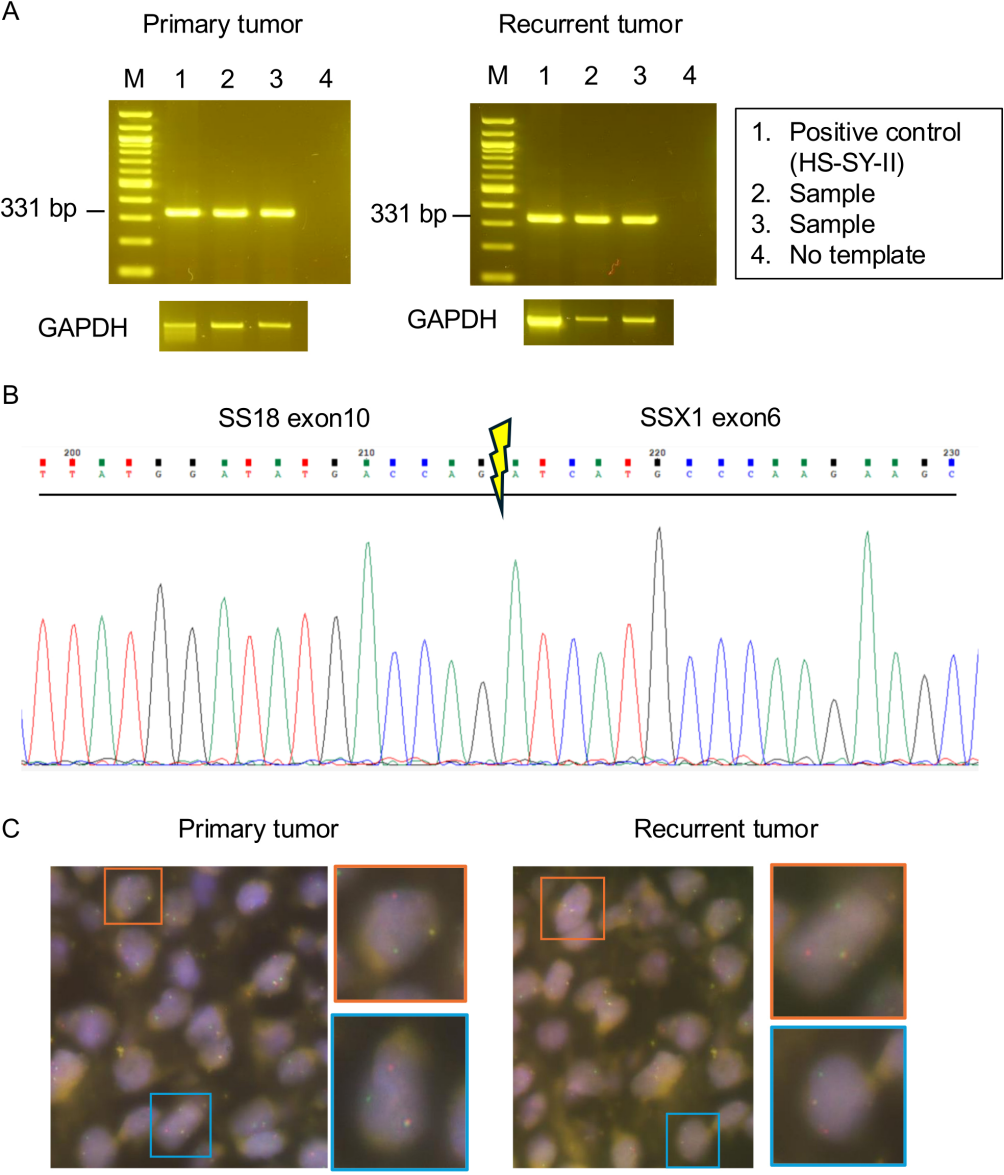
Molecular analysis of the tumor. Reverse transcription polymerase chain reaction (RT-PCR) performed on both the resected primary tumor and the recurrent subcutaneous tumor demonstrated amplification of the SS18–SSX1 fusion transcript **(A)**. Sanger sequencing revealed the canonical fusion of SS18 exon 10 to SSX1 exon 6 **(B)**. Fluorescence *in situ* hybridization (FISH) revealed SS18 split signals in tumor cells from both specimens **(C)**.

Histologically, the biopsy specimen exhibited proliferation of short spindle to round tumor cells in a faintly basophilic myxoid stroma, accompanied by nuclear atypia with minimal pleomorphism (**Figure 2A**). Immunohistochemically, the tumor cells were diffusely positive for vimentin (**Figure 2B**), SOX9 (**Figure 2C**), and CD56 (**Figure 2D**), and focally positive for CDK4. The tumor cells were negative for desmin, SMA, S100, CD34, CD99, MDM2, synaptophysin, chromogranin, CD117, and cytokeratin (keratin mix) (**Figure 2E**). The Ki-67 labeling index was approximately 20% (**Figure 2F**). The immunohistochemical results for both the biopsy specimen and the resected tumor are summarized in **Supplementary Table 2**. Based on these findings, the lesion was interpreted as a myxoid neoplasm, and the differential diagnosis included EMC, myxoid liposarcoma, myxofibrosarcoma, undifferentiated pleomorphic sarcoma, and myxoma. Synovial sarcoma was not initially suspected, and therefore molecular testing for SS18 rearrangement was not performed at that stage. Since HE staining and SOX9 positivity suggested EMC, fluorescence *in situ* hybridization (FISH) for EWSR1 was performed; however, no split signals were detected. The resected tumor exhibited intratumoral hemorrhage and necrosis (**Figure 3A**), with proliferation of short spindle-shaped to round tumor cells in the myxoid stroma. Histologically, these findings were consistent with those of the biopsy specimens (**Figures 3B, C**). Immunohistochemically, the tumor cells were diffusely positive for vimentin, SOX9, and CD56 and negative for cytokeratin (AE1/AE3), EMA, S100, c-kit, GFAP, CD99, synaptophysin, chromogranin, desmin, CD34, INSM1, and BCL2 (**Supplementary Table 2**; **Figures 3D, E**). A hemangiopericytoma-like vascular pattern was only focally and inconspicuously present and did not represent a prominent or defining feature of the tumor. Calcifications were not identified, and mast cell infiltration was not evident. The surgical margin was positive, and lymphovascular invasion involving the femoral vein was present, whereas perineural invasion was absent. Although a definitive diagnosis was not established at the time of the initial surgery, considering the final diagnosis of synovial sarcoma, the tumor showed a mitotic index of 10–19 mitoses per 10 high-power fields, corresponding to FNCLCC grade 3. Histological findings of the resected recurrent subcutaneous tumor were consistent with those of both the biopsy specimen and the resected primary tumor (**Supplementary Figure 2**). In focal areas, the tumor cells were spindled with eosinophilic cytoplasm and arranged in a reticular pattern, resembling EMC (**Supplementary Figure 3**). The resected recurrent subcutaneous tumor was subjected to comprehensive genomic profiling (CGP), which identified an SS18–SSX1 fusion transcript joining SS18 exon 10 to SSX1 exon 6, with abundant junction-spanning reads and no additional pathogenic gene fusions (GSE324018). To further confirm the diagnosis, reverse transcription polymerase chain reaction (RT-PCR) was performed on both the resected primary tumor and the recurrent subcutaneous tumor as previously described (7), and amplification of the SS18–SSX1 fusion transcript was detected (**Figure 4A**). The RT-PCR product was subsequently confirmed by Sanger sequencing, revealing the canonical fusion of SS18 exon 10 to SSX1 exon 6, consistent with previous reports (7, 8) (**Figure 4B**). Additionally, FISH was performed on both specimens, which demonstrated SS18 split signals in each tumor (**Figure 4C**). We did not perform histological evaluation of the pulmonary lesion; however, based on radiological progression, it was clinically considered to represent pulmonary metastasis.

## 3 Discussion

SS is a translocation-associated sarcoma defined by SS18–SSX fusions, and most cases exhibit immunohistochemical focal epithelial differentiation. However, SS can exhibit a broad stromal spectrum, including rare tumors with extensive myxoid change (4, 9–12). In the present case, the neoplasm exhibited diffuse myxoid stroma, SOX9 positivity, and epithelial markers negativity, which collectively prompted consideration of EMC at the initial work-up. CGP subsequently identified an SS18–SSX1 fusion, leading to the molecular confirmation of SS in the primary and recurrent tumors. To our knowledge, this is the first reported case of synovial sarcoma arising in the extremity that exhibits diffuse myxoid stroma together with a complete absence of epithelial marker expression, with the diagnosis confirmed by molecular identification of the specific fusion transcript.

A distinctive feature of the present tumor was its diffuse myxoid background. Although myxoid-predominant cases account for only about 2% of all SSs, myxoid SS has been recognized as an uncommon morphological subset that may obscure the classic appearance of SS and cause substantial diagnostic difficulty (4). In contrast to the usual presentation, in which myxoid change is typically focal (4), this case exhibited predominantly diffuse myxoid histology. Because of the abundant myxoid stroma, the initial differential diagnosis included various myxoid tumors, such as EMC, myxoid liposarcoma, myxofibrosarcoma, undifferentiated pleomorphic sarcoma with myxoid change, and even benign myxoid tumors; however, synovial sarcoma was not considered. As a result, SS was not initially included in the diagnostic work-up. In retrospect, if the possibility of myxoid change in SS had been considered, SS would have been considered in the differential diagnosis, particularly given the relatively low degree of pleomorphism observed in this tumor. Furthermore, retrospective review of this case identified only subtle features suggestive of SS, such as a hemangiopericytoma-like vascular pattern. The absence of scattered mast cells and focal calcification or ossification further complicated the diagnosis. Consistent with this, previous reports have emphasized that myxoid-predominant SS may be mistaken for other myxoid sarcomas, particularly when typical epithelial marker expression is absent or minimal (4). Taken together, these findings highlight that synovial sarcoma may exhibit atypical morphological features, such as myxoid change, and that this possibility should be considered during diagnostic evaluation.

The second key diagnostic pitfall in this case was SOX9 immunoreactivity. Although SOX9 is widely recognized as a transcription factor involved in chondrogenesis and is often interpreted as supporting a chondroid lineage, its expression is not specific to chondroid tumors. In a broad immunohistochemical survey, the majority of SSs (16/18; 88.9%) showed moderate to strong nuclear SOX9 staining, indicating that SOX9 positivity can be misleading in the differential diagnosis of bone and soft tissue tumors (13). In this case, diffuse SOX9 positivity together with prominent myxoid stroma led to an initial diagnosis of a myxoid tumor with chondroid differentiation, with EMC considered the leading possibility. Taken together, SOX9 positivity should not be regarded as specific for chondroid tumors, and interpretation of SOX9 positivity should be integrated with morphological features and molecular findings to avoid diagnostic misclassification.

In this patient, EMC was considered the leading diagnosis based on the HE and immunohistochemical findings. Accordingly, FISH for EWSR1, which is available at our institution, was performed to investigate the presence of the EWSR1–NR4A3 fusion gene, which is the most common fusion in EMC. However, several alternative fusion partners of NR4A3 have been reported in EMC; therefore, assessment of EWSR1 split signals alone is insufficient to exclude EMC. Thus, EMC could not be readily ruled out even in the absence of EWSR1 split signals.

Epithelial markers are useful in most cases of SS; however, rare epithelial marker–negative cases have been reported in SS of the kidney (11) and the metastatic case of unknown primary (14), with the diagnosis confirmed by detection of the SS18–SSX fusion. In addition, a case of synovial sarcoma arising in the foot without epithelial marker expression has been reported based on SS18 rearrangement, even though direct identification of the SS18–SSX fusion transcript was not demonstrated (15). Moreover, caution should be exercised when interpreting the immunohistochemical detection of epithelial differentiation in SS. Although more than 90% of cases may show at least focal positivity for cytokeratin or epithelial membrane antigen (EMA), discordant expression (cytokeratin-positive/EMA-negative or vice versa) has been documented, and combined assessment of both markers is recommended (16). In the present case, both cytokeratin and EMA were examined but were negative, which contributed to the diagnostic difficulty. Accordingly, molecular confirmation remains the gold standard when immunophenotypes are confusing (16). Although a rare “dual-fusion” tumor with combined SS and EMC features has been reported (12), RNA sequencing in our case revealed no additional pathogenic fusion genes, indicating that the tumor was driven by SS18–SSX1 alone. In addition, rare SS18-negative variant fusions have been identified in synovial sarcoma, including EWSR1–SSX1 and other non-canonical SSX1 fusions (17, 18), indicating that synovial sarcoma cannot always be diagnosed based solely on SS18 break-apart FISH. Pathologists should be aware of these atypical rearrangements when molecular findings are discordant with morphology.

Recent real-world data supports the clinical utility of CGP in sarcomas. In a cohort of 136 patients with sarcoma undergoing CGP testing in routine practice in Japan, the detection of histology-specific translocations led to diagnostic reclassification in 2.2% of the cases (19). In another study, genomic profiling resulted in diagnostic revision in 12 of 83 patients (14%) with sarcoma, and in 8 of these cases, the treatment strategy was subsequently modified (20). In the present case, the CGP identified SS18–SSX1 and enabled subsequent confirmatory testing, highlighting the value of fusion-oriented genomic testing in diagnostically challenging cases. Although fusion panel testing is a highly useful modality, it requires a longer turnaround time compared with targeted assays, and evidence supporting its non-selective, routine use in patients with sarcoma remains limited at present (21). Further investigation is needed to determine the optimal timing of CGP testing in such cases.

From a clinical perspective, in sarcomas, both chemotherapy-sensitive and -insensitive tumors exist; SS is generally considered sensitive to chemotherapy, whereas EMC is regarded as relatively insensitive (6). Although the role of neoadjuvant and adjuvant chemotherapy in soft tissue sarcomas has not yet been definitively established, accumulating evidence in recent years has supported its clinical benefit (6). Therefore, establishing an accurate pathological diagnosis is crucial, as it directly impacts clinical management, including decisions regarding the use of systemic therapy. In the present case, given the initial diagnostic prioritization of EMC, SS18 break-apart FISH or a broader fusion panel was not performed at an early stage. In retrospect, this represents a missed diagnostic opportunity, as earlier molecular testing for SS might have facilitated a more timely and accurate diagnosis. In this case, considering the invasiveness of the procedure and the patient’s activities of daily living, the indication for a wider resection and the use of neoadjuvant and adjuvant chemotherapy remain debatable; however, in general, earlier establishment of the correct diagnosis can influence clinical management, including consideration of a wider surgical margin and more tailored adjuvant therapy. Previous reports have also emphasized that molecular genetic testing is essential for improving diagnostic accuracy in sarcomas and ensuring appropriate clinical management (22). Considering these findings, in diagnostically challenging soft tissue tumors with atypical morphology, a broader molecular diagnostic approach, including fusion testing (e.g., FISH, RT-PCR, or CGP), should be considered early in the diagnostic process, even when a specific sarcoma subtype is not initially suspected.

In this case, three cycles of doxorubicin monotherapy resulted in progressive disease. Therefore, pazopanib was selected as second-line therapy after discussion with the patient. Following initiation of pazopanib, tumor growth has been limited to a slight increase, and the treatment is currently ongoing. Pazopanib has been shown to improve progression-free survival in previously treated patients with advanced soft tissue sarcoma, including synovial sarcoma (23), which is consistent with the present clinical course. Although this case is characterized by diffuse myxoid stroma and complete absence of epithelial marker expression, there is currently no established evidence that these features influence chemosensitivity or the selection of targeted therapies, and further investigation is warranted. Regarding fusion subtype and prognosis, most studies have compared SS18–SSX1 and SS18–SSX2. Some reports suggest that SS18–SSX1 is associated with a worse prognosis than SS18–SSX2 (24–26), whereas others have found no significant prognostic difference (27, 28). A recent systematic review and meta-analysis reported no significant differences in overall survival or disease-specific survival between the two subtypes; however, SS18–SSX1 was associated with unfavorable outcomes in terms of progression-free survival and metastasis-free survival (29).

## 4 Conclusions

In summary, SS with a prominent myxoid stroma may mimic other myxoid sarcomas and pose a significant diagnostic challenge, particularly in the absence of epithelial markers. This case highlights the importance of integrating morphological, immunohistochemical, and molecular analyses. Fusion-oriented genomic testing, such as CGP, can play a decisive role in establishing the correct diagnosis of diagnostically challenging sarcomas.

## Data Availability

The datasets presented in this study can be found in online repositories. The names of the repository/repositories and accession number(s) can be found in the article/**Supplementary Material**.

## Supplementary material

Supplementary Figure 1 MRI findings of the primary tumor in the thigh. The mass in the right thigh showed iso- to partially hyperintense signal on T1-weighted images **(A)**, heterogeneous hyperintensity on T2-weighted images **(B)**, and partial contrast enhancement on gadolinium-enhanced T1-weighted images **(C)**.

Supplementary Figure 2 Histological findings of the recurrent tumor. Hematoxylin and eosin staining showed short spindle to round tumor cells proliferating in a myxoid stroma **(A, B)**. Original magnification ×40 for **(A)** and ×200 for **(B)**.

Supplementary Figure 3 EMC-like lesion in the resected tumor. The resected tumor focally showed a morphology resembling EMC, with spindled tumor cells with eosinophilic cytoplasm arranged in a reticular pattern. Original magnification: ×40 **(A)**, ×100 **(B)**, and ×200 **(C)**.

Supplementary Table 1 Clinical course of the patient. –, none; CGP, comprehensive genomic profiling.

Supplementary Table 2 Immunohistochemical findings in the biopsy and resected specimens of the primary tumor. +, positive; −, negative; N/T, not tested.
