# Supplementary figures and images for: Case Report: Synovial sarcoma with diffuse myxoid stroma and complete absence of epithelial differentiation in the extremity
Source: Front Oncol. 2026 May 29;16:1846272. doi: 10.3389/fonc.2026.1846272 (PMC13259740; doi:10.3389/fonc.2026.1846272)

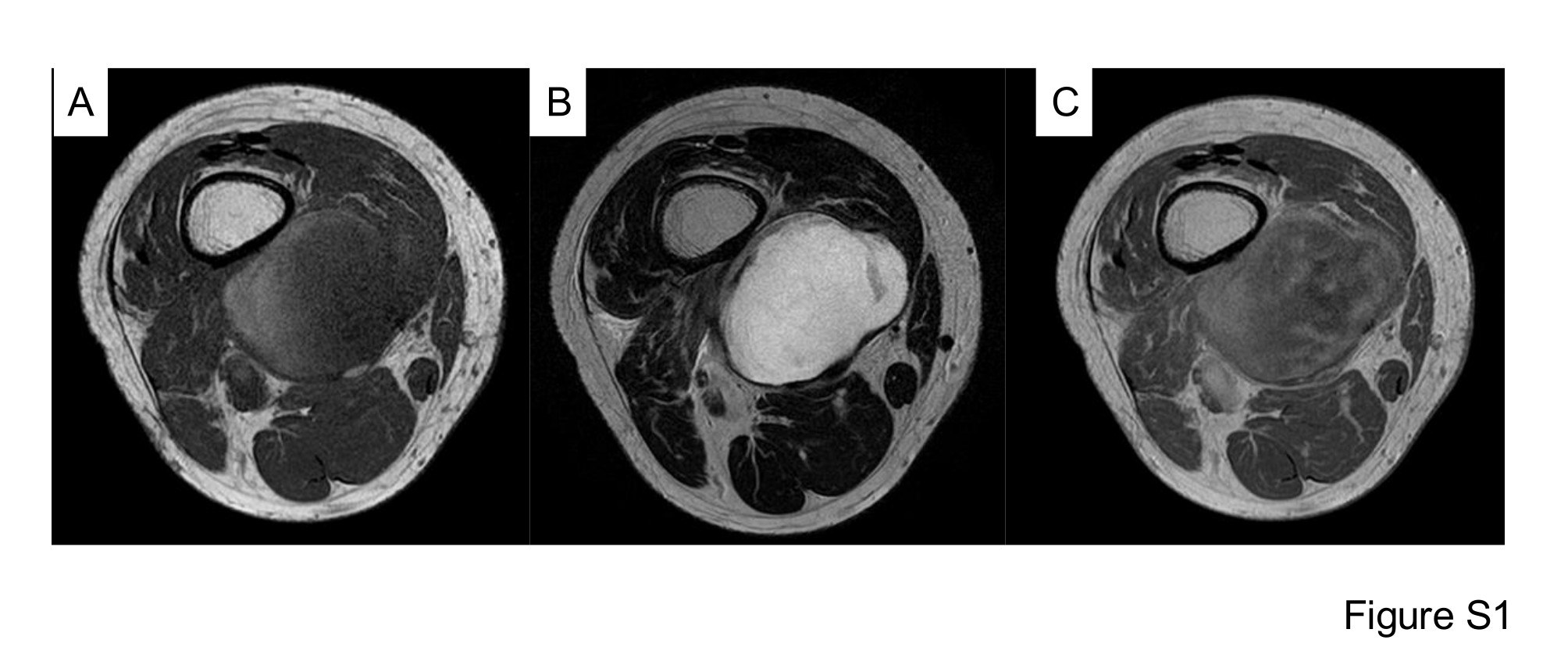

Supplement: Supplementary Figure 1 — MRI findings of the primary tumor in the thigh. The mass in the right thigh showed iso- to partially hyperintense signal on T1-weighted images (A), heterogeneous hyperintensity on T2-weighted images (B), and partial contrast enhancement on gadolinium-enhanced T1-weighted images (C). [file Image1.jpeg]

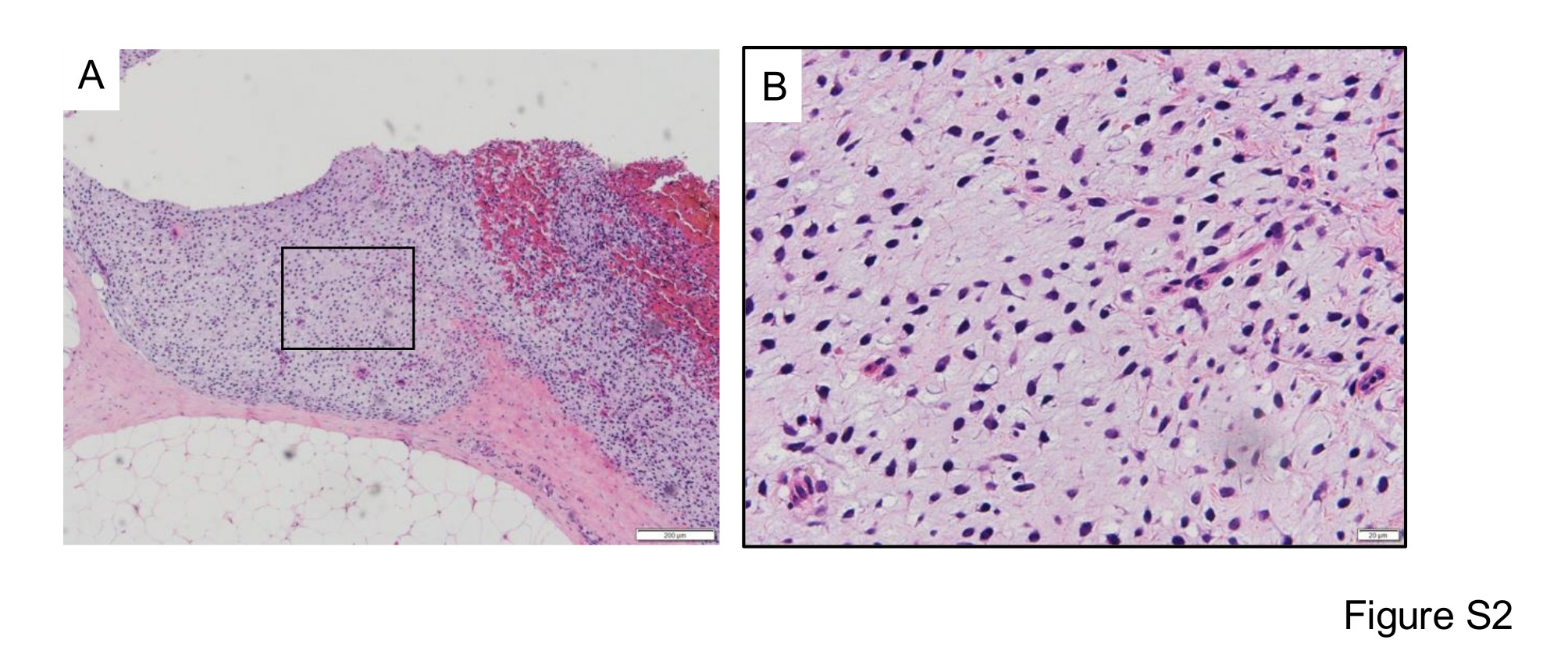

Supplement: Supplementary Figure 2 — Histological findings of the recurrent tumor. Hematoxylin and eosin staining showed short spindle to round tumor cells proliferating in a myxoid stroma (A, B). Original magnification ×40 for (A) and ×200 for (B). [file Image2.jpeg]

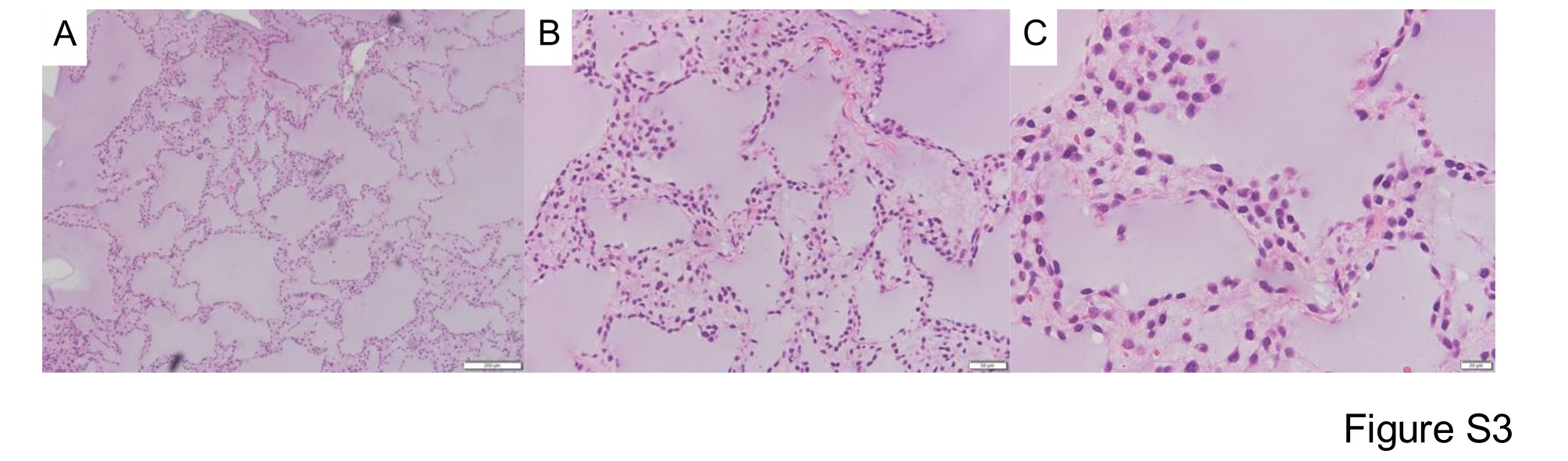

Supplement: Supplementary Figure 3 — EMC-like lesion in the resected tumor. The resected tumor focally showed a morphology resembling EMC, with spindled tumor cells with eosinophilic cytoplasm arranged in a reticular pattern. Original magnification: ×40 (A), ×100 (B), and ×200 (C). [file Image3.jpeg]
