# Supplementary material for: Case Report: Synovial sarcoma with diffuse myxoid stroma and complete absence of epithelial differentiation in the extremity
Source: Front Oncol. 2026 May 29;16:1846272. doi: 10.3389/fonc.2026.1846272 (PMC13259740; doi:10.3389/fonc.2026.1846272)
Supplement: Supplementary Table 1 — Clinical course of the patient. –, none; CGP, comprehensive genomic profiling. [file Table1.docx]

Supplementary Table 1. Clinical course of the patient

| **Time** | **Clinical events** | **Diagnostic findings** | **Treatment** |
| --- | --- | --- | --- |
| Initial visit | Palpable thigh mass | MRI: 55-mm tumor | – |
| Month 0 | Biopsy | Myxoid tumor suspected | – |
| Month 1 | Surgery | – | Marginal excision + vein resection |
| Month 1–2 | – | – | Radiotherapy (60 Gy in total) |
| Month 15 | – | Small left apical pulmonary nodule detected; indeterminate, continued radiological follow-up | – |
| Month 25 | – | Slowly enlarging pulmonary nodule; metastasis strongly suspected | Pulmonary resection and systemic therapy discussed; patient opted for observation |
| Month 40 | Recurrence | Local recurrence and pulmonary progression | – |
| Month 41 | Reoperation | – | Resection of recurrent subcutaneous lesion; CGP performed |
| Month 42–44 | – | – | Doxorubicin (3 cycles); progressive disease |
| Month 45–present | – | – | Pazopanib initiated; tumor progression relatively slow; treatment ongoing |

–, none; CGP, comprehensive genomic profiling.
