# Supplementary material for: Case Report: Synovial sarcoma with diffuse myxoid stroma and complete absence of epithelial differentiation in the extremity
Source: Front Oncol. 2026 May 29;16:1846272. doi: 10.3389/fonc.2026.1846272 (PMC13259740; doi:10.3389/fonc.2026.1846272)
Supplement: Supplementary Table 2 — Immunohistochemical findings in the biopsy and resected specimens of the primary tumor. +, positive; −, negative; N/T, not tested. [file Table2.docx]

Supplementary Table 2. Immunohistochemical findings in the biopsy and resected specimens of the primary tumor

| Category | Marker | Biopsy | Resection |
| --- | --- | --- | --- |
| Mesenchymal markers | Vimentin | + | + |
|  | SOX9 | + | + |
| Neural markers | CD56 | + | + |
| Muscle markers | Desmin | − | − |
|  | SMA | − | N/T |
| Melanocytic markers | S100 | − | − |
| Epithelial markers | AE1/AE3 | N/T | − |
|  | Keratin mix | − | N/T |
|  | EMA | N/T | − |
| Neuroendocrine markers | Synaptophysin | − | − |
|  | Chromogranin | − | − |
|  | INSM1 | N/T | − |
| Other markers | MDM2 | − | N/T |
|  | CDK4 | + (focal) | N/T |
|  | CD34 | − | − |
|  | KIT (CD117) | − | − |
|  | GFAP | − | − |
|  | CD99 | N/T | − |
|  | BCL2 | N/T | − |
| Special stain | Alcian blue | N/T | + |
| Proliferation | Ki-67 index | about 20% | about 10% |

+, positive; −, negative; N/T, not tested.
